# Amino Acid Chiral Selection Via Weak Interactions in Stellar Environments: Implications for the Origin of Life

**DOI:** 10.1038/s41598-018-27110-z

**Published:** 2018-06-11

**Authors:** Michael A. Famiano, Richard N. Boyd, Toshitaka Kajino, Takashi Onaka, Yirong Mo

**Affiliations:** 1 Department of Physics and Joint Institute for Nuclear Astrophysics, Western Michigan Univ., 1903 W. Michigan Avenue, Kalamazoo, MI 49008-5252 USA; 2 National Astronomical Observatory of Japan, 2-21-1 Mitaka, Tokyo, 181-8588 Japan; 3 Department of Physics, Department of Astronomy, The Ohio State Univ., Columbus, OH 43210 USA; 4 Department of Astronomy, Graduate School of Science, Univ. of Tokyo, 7-3-1 Hongo, Bunkyo-ku, Tokyo, 113-0033 Japan; 5 School of Physics and Nuclear Energy Engineering, Beihang Univ. (Beijing Univ. of Aeronautics and Astronautics), Beijing, 100083 P.R. China; 6 Department of Chemistry, Western Michigan Univ., 1903 W. Michigan Avenue, Kalamazoo, MI 49008-5252 USA

## Abstract

Magnetochiral phenomena may be responsible for the selection of chiral states of biomolecules in meteoric environments. For example, the Supernova Amino Acid Processing (SNAAP) Model was proposed previously as a possible mode of magnetochiral selection of amino acids by way of the weak interaction in strong magnetic fields. In earlier work, this model was shown to produce an enantiomeric excess (*ee*) as high as 0.014% for alanine. In this paper we present the results of molecular quantum chemistry calculations from which *ee*s are determined for the *α*-amino acids plus isovaline and norvaline, which were found to have positive *ee*s in meteorites. Calculations are performed for both isolated and aqueous states. In some cases, the aqueous state was found to produce larger *ee*s reaching values as high as a few percent under plausible conditions.

## Introduction

Recent work on the SNAAP Model of enantiomeric excess (*ee*) production in amino acids^1–6^ has been shown^6^ to produce large enough *ee*s to allow it to explain how amino acids were created in space and observed in meteorites. This model is initiated via weak interactions in amino acids in strong magnetic and electric fields. After an initial enantiomeric excess is created, subsequent autocatalysis and processing could be responsible for relative abundances observed in meteorites^7–13^. We define the enantiomeric excess, $ee( \% )=\frac{{N}_{L}-{N}_{D}}{{N}_{L}+{N}_{D}}\times 100 \%$, where N_*L*_ and N_*D*_ represent, respectively, the numbers of left- and right-handed amino acids in any ensemble. In this paper we present results for determining the *ee*s for 21 amino acids, including proteinogenic *α*-amino acids (those in which the amine group is bonded directly to the *α* - or central - carbon atom), as well as isovaline and norvaline. It was shown from meteoritic analyses^13^ that *ee*s may undergo processing in an aqueous environment. Thus, we have performed calculations for these amino acids both in covalent (gas phase) and zwitterionic (aqueous or crystalline) forms, with a few notable examples computed for the amino acids in their cationic or anionic forms. These include histidine, asparagine, and lysine, which are cationic in solution, as well as aspartic and glutamic acids, which are anionic in solution. In either case, the amino acids could be in an aqueous environment.

Following the formation of an amino acid enantiomeric excess in meteorites, these meteorites may have then seeded the primordial earth. The encased amino acids would have undergone autocatalysis either prior to terrestrial impact or during subsequent processing on the early earth. Autocatalytic mechanisms have been postulated to couple a small enantiomeric excess to homochirality. In some models, the pre-existing chiral bias would have been destroyed in favor of a chiral bias produced in the primordial environment^14^. This and other models may have been enhanced or implemented via statistical fluctuations in the environment with enough amplitude to drive the environment to a specific chirality^15,16^.

In any case, the origin of amino acid enantiomeric excesses - whether terrestrial or extra-terrestial - has profound implications for the origin of life on earth.

The details of the SNAAP model, including the mathematical analysis, were presented in earlier publications^1,2,6^, so only its basics will be presented here. We will then give some of the details of the calculations and present the results and conclusions.

## The Supernova Neutrino Amino Acid Processing Model

In the SNAAP model, described in detail previously^3,6^, it is assumed that the amino acids are produced and constrained in meteoroids, since this has been found to be the case for those that made their way to Earth^7–13^. When a supernova explodes, whatever meteoroids are in the vicinity of the nascent neutron star will be subjected to the strong magnetic field that results from the core collapse and to an intense flux of electron antineutrinos that cool the star. Another possible site^17^ is a close binary system comprised of a neutron star and Wolf-Rayet star. An accretion disk would be formed from the material attracted from the WR star to the neutron star. The material in the disk would be processed by the magnetic field from the neutron star, the electric field produced by the motion of meteoroids in the disk through the magnetic field, and the neutrinos from the cooling neutron star or from the supernova.

The electron antineutrinos, hereafter denoted as “antineutrinos,” will interact differently with the nitrogen nuclei in the amino acids depending on whether the spin of the ^14^N (which has a spin = 1 in units of Planck’s constant divided by 2*π*) is aligned or antialigned with that of the (spin 1/2) antineutrinos. The relevant reaction is ${\bar{\nu }}_{e}+{}^{14}N\to {}^{14}C+{e}^{+}$. From basic weak interaction nuclear physics it is known^18^ that the cross section for this reaction if the spins are aligned is about an order of magnitude less than if the spins are antialigned, since in the former case, conservation of angular momentum requires that the wave function of either the ${\bar{\nu }}_{e}$ or the *e*^+^ provides one unit of angular momentum. This, along with the orientation of the ^14^N spin in the magnetic field, produces a selective destruction of ^14^N in one orientation.

Although the *ee*s in any model of amino acid production are smaller than some of those observed in meteorites, it is generally accepted that autocatalysis^19–22^ will drive the small amino acid *ee*s to the values observed, and ultimately to Earthly homochirality.

In the previous work^6^, several effects were investigated that would couple the N spin to the alanine molecule. It was shown that the selective destruction of the N would indeed produce an *ee*, which was found to be dominated by the left-handed chirality observed in meteorites. The maximum *ee* found was 0.014%. For the present paper we describe calculations for the amino acids in covalent, zwitterionic, cationic, and anionic geometries. Zwitterionic forms were found to produce considerably larger *ee*s in most *α*-amino acids.

It is assumed in the SNAAP model that the relevant objects being processed in the field of the supernova explosion would be large enough that some surface material could be ablated away upon entry into the atmosphere, and a meteoroid of a smaller size would remain. However, this object would preserve the *ee* that the original larger object would have had. Because the interaction cross section for neutrinos to interact with matter is so small^23^, many neutrinos would be able to penetrate to the centers of bodies of any size. The extremely large neutrino flux associated with supernova events and neutron stars is what enables a reasonable interaction rate of neutrinos incident on the body.

The *ee* produced by such a mechanism would not be altered by ablation heating during atmospheric entry except on the surface.

A critical feature of the SNAAP Model^6^ is the coupling of the nuclear spin to the molecular chirality. The effects of the molecular electronic orbitals and molecular orientation are linked to the nuclear spin via the nuclear magnetic shielding tensor, *σ*. Differences in the shielding tensor from different molecular chiral states can affect the magnetic fields at the nucleus, and thus the overall spin orientations of the ^14^N nuclei with respect to the external field. In this model, external fields can implement this shift via the “Buckingham Effect”^24–26^. Because the mathematics associated with the nuclear-molecular coupling is lengthy, we will refer the interested reader to reference^6^.

## Calculations

In a previous paper^6^, the values of the nuclear magnetic polarizabilities of L- and D-alanine were calculated following the example of reference^25^. In the current work, the Gaussian quantum chemistry code^27^ was used to compute the asymmetric shielding tensor. Previously, the polarizability was determined via a calculation of the tensor in the presence of external electric fields, which were treated perturbatively.

Here, we have computed the asymmetric shielding tensors for the *α*-amino acids in both ligand and zwitterionic form. Electron orbital wave functions were optimized in the aug-cc-pVDZ basis^28^ using a second-order Møller-Plesset (MP2) calculation^29^ followed by tensor determinations using these orbitals in a DFT calculation with the B3LYP hybrid functional^30^ and the pcS-2 basis set^31,32^.

In a coordinate system with axes defined by the fields, the molecular electric dipole moment results in a preferred temperature-dependent orientation of the molecule with respect to the external electric field and thus a preferred average orientation with respect to the external magnetic field. The shielding tensor components corresponding to the external fields result in chirality-dependent shifts of the magnetic field at the nucleus. *That is*, *the molecular orientation is controlled by the external electric field*, *the shielding tensor is affected by the molecular orientation in the magnetic field*, *and the nuclear orientation is affected by the resulting local magnetic field*.

### Effects From the Molecular Electric Dipole Moment

The influence of the molecular polarization in an electric field is described below. Because this model has been described previously^6^, we describe the main points here, referencing the vector diagram in Fig. 6 from Famiano *et al*.^6^.

The electric dipole moment depends on the molecular shape, so it is also chiral. In the presence of electric fields, populations of field-aligned molecules follows a Maxwell-Boltzmann distribution^26,33^; molecular electronic orbitals have a preferred orientation in the external electric field. Likewise, the nuclei have a preferred orientation in the external magnetic field. The molecular motion in the external magnetic field then results in a perpendicular electric field. Thus, average nuclear orientation can be established relative to the molecular orientation. Because of the molecular orientation in the external fields, a preferred coordinate system is created for the shielding tensor; it is not isotropic. For an electric dipole moment *μ*_*E*_, a nuclear magnetization **M**, and an electric field **E**, the nuclear magnetization shift Δ**M**_*T*_ is^26,33^:

1 $$\begin{array}{ccc}{{\rm{\Delta }}}_{T}{\bf{M}} & = & \frac{1}{6kT}[({\sigma }_{xy}-{\sigma }_{yx}){\mu }_{E,z}\\  &  & +({\sigma }_{yz}-{\sigma }_{zy}){\mu }_{E,x}+({\sigma }_{zx}-{\sigma }_{xz}){\mu }_{E,y}]({\bf{M}}\times {\bf{E}})\\  & = & \frac{{\bf{M}}\times {\bf{E}}}{6kT}{\eta }_{M}\end{array}$$

where the “molecular geometry factor” *η*_*M*_ depends solely on the molecular properties:

2 $${\eta }_{M}\equiv ({\sigma }_{xy}-{\sigma }_{yx}){\mu }_{E,z}+({\sigma }_{yz}-{\sigma }_{zy}){\mu }_{E,x}+({\sigma }_{zx}-{\sigma }_{xz}){\mu }_{E,y}$$

Thus the shift in magnetization Δ**M** can be written as a product of a molecular-dependent term, an external environmental-dependent term (temperature and electric field), and the nuclear term **M**. In this model, the magnetization is perpendicular to the electric field, and the relative shift in magnetization is then:

3 $$\frac{{\rm{\Delta }}M}{M}=\frac{E}{6kT}{\eta }_{M}$$

The term *η*_*M*_ results in a shift in magnetization parallel to the meteoroid velocity in an external magnetic field. For *η*_*M*_ > 0, Δ*M* is in the direction of the meteoroid velocity, and for *η*_*M*_ < 0, Δ*M* is opposite to the meteoroid velocity. Since *η*_*M*_ is chirality-dependent, the resultant magnetization, **M** + Δ**M**, is chirality dependent, and makes an angle *ϕ* with respect to the external magnetic field:

4 $$\begin{array}{rcl}{\tan }^{-1}\,\varphi  & = & \frac{{\rm{\Delta }}M}{M}=\frac{E}{6kT}{\eta }_{M}\\ \to \varphi  & \approx  & \frac{E}{6kT}{\eta }_{M}\end{array}$$

A larger value of *η*_*M*_ will result in a larger angle, and a greater difference in the component of spins parallel and anti-parallel to the spins of the antineutrinos exiting the neutron star. This will result in a larger *ee*. The sign of the *ee* will depend on the value of ${\bf{M}}\times {{\bf{E}}}_{TS}\cdot {{\bf{v}}}_{\bar{\nu }}$ and *η*_*M*_, where ${{\bf{v}}}_{\bar{\nu }}$ is the antineutrino velocity vector, and **E**_*TS*_ is the electric field vector in the meteoroid’s rest frame; if both have the same sign, then *ee* > 0 (See Fig. 6 of Famiano *et al*.^6^).

The above is a result of the thermally averaged electrical polarization of the molecule oriented in a static external electric field. In this case, the anisotropic components of the shielding tensor times the electric dipole moment vector results in a non-zero contribution to the change in magnetization. This non-isotropic effect can exceed the isotropic effects of shielding tensor by several orders of magnitude^25^.

If the meteoroid is not moving with respect to the magnetic field, the bulk magnetization is **M**. For a non-zero meteoroid velocity, an additional transverse magnetization component, Δ**M**_*L*,*D*_, is created where the subscript indicates the L- or D- chirality. The chirality-dependent magnetization creates a shift in the nuclear spin vectors with populations *N*_±,*L*/*D*_. The magnetization shift then results in an angle between the nitrogen spin and the antineutrino spin which is chirality-dependent. This means the reaction rates between antineutrinos and nitrogen vary with chirality, because nitrogen in each chiral state has a different spin alignment.

The reaction rate ratio for antineutrino spins parallel and anti-parallel to the ^14^N spin component is estimated^6^:

5 $$\frac{{R}_{p}}{{R}_{a}}=\frac{1-\,\cos \,{{\rm{\Theta }}}_{p}}{1-\,\cos \,{{\rm{\Theta }}}_{a}}=\frac{1-\,\sin \,\varphi }{1+\,\sin \,\varphi }\approx \frac{1-\varphi }{1+\varphi }\to \frac{1-\frac{E}{6kT}{\eta }_{M}}{1+\frac{E}{6kT}{\eta }_{M}}.$$

For the situation described, a positive *η*_*M*_ will result in a positive *ee* as the destruction rates for L-amino acids are smaller. (For 90° < *ϕ* < 180°, a negative value of *η*_*M*_ will result in an excess of L-amino acids.)

With the above destruction rate ratios the time-dependent spin-state populations for each chirality, *N*_±,*D*/*L*_, and the enantiomeric excess of a particular state can now be computed.

6 $$ee( \% )=\frac{({N}_{+,L}+{N}_{-,L})-({N}_{+,D}+{N}_{-,D})}{{N}_{+,L}+{N}_{-,L}+{N}_{+,D}+{N}_{-,D}}\times 100 \% $$

## Results and Conclusions

The value of *η*_*M*_ was computed for both the ligand and zwitterionic forms. Ligand geometries were taken from the Protein Data Bank^34^, and zwitterionic geometries were taken from the Cambridge Structural Database^35^.

The relative destruction rate, and hence the resultant *ee*, depends on the geometry factor *η*_*M*_. The computed values of *η*_*M*_ are shown in Table 1 for both geometries (units in ppm-a.u.). In this table, cation values are in bold, and anion values are underlined. Values separated by commas indicate two geometries in the same reference. Though the ligand geometries generally result in small values of *η*_*M*_ of both signs, the zwitterionic geometry generally produces positive *η*_*M*_ owing to the larger electric dipole moment of the zwitterionic form.

**Table 1:** Complete table of the molecular geometry parameter *η*_*M*_ for amino acids not mentioned in the text.

Amino Acid	Ligand	Zwitterion	Optimized	*EE*_*max*_(×10^−6^)	References
Alanine	−3.87	31.79	39.39	2.52	LALNIN59^47^
			**51**.**60**	**3**.**35**	XIYSAA^48^
Arginine	7.79	−44.11	−160.41	−10.4	TAQBIY^49^
		**18**.**57**, **47**.**18**		**1**.**2**	ARGHCL11^50^
Asparagine	21.88	0.05	−36.39	−2.36	VIKKEG^51^
		38.33	37.53	2.38	ASPARM09^52^
Aspartic Acid	11.22	11.83	23.66	1.53	LASPRT04^53^
		15.14		0.98	NAGLYB10^54^
Cysteine	10.24	8.92	10.60	0.688	LCYSTN36^55^
Glutamic Acid	0.94	28.48	26.54	1.72	LGLUAC03^56^
		153.95		10.00	CAGLCL10^57^
Glutamine	−4.76	16.43	9.22	0.60	GLUTAM02^58^
Histidine	−10.55	−44.58	−31.20	−2.02	LHISTD13^59^
		**20**.**21**		**1**.**31**	HISTCM12^60^
Isoleucine	5.67	−5.24, 28.00	17.58	1.14	LISLEU02^61^
Isovaline	−0.63	−1.92	−16.67	−1.08	KIMKUO^62^
			**119**.**94**	**7**.**68**	
Leucine	1.95	34.78	30.91	2.01	LEUCIN04^63^
Lysine	0.53	0.09, 42.92	19.78	1.28	CUFFUG^64^
		**−14**.**82**		**−0**.**96**	LYSCLH11^65^
Methionine	−1.52	−0.34, 19.09	26.73	1.74	LMETON14^66^
Norvaline	5.49	26.24	33.22	2.16	USOHUH04^66^
			**10**.**50**	**0**.**68**	VUKQID^67^
Phenylalanine	12.10	19.3	21.15	1.37	QQQAUJ07^68^
Proline	−3.68	17.5	47.25	3.07	PROLIN01^69^
Serine	6.84	11.83	13.53	0.88	LSERIN28^70^
Threonine	−6.43	12.20	−6.24	−0.41	LTHREO04^47^
Tryptophan	18.02	6.51	1.59	0.10	VIXQOK01^71^
Tyrosine	19.72	28.37	−8.90	−0.57	LTYROS11^72^
Valine	1.01	4.44, 34.52	19.87	1.29	LVALIN05^73^
			**8**.**47**	**0**.**55**	VALEHC11^74^

Also included are geometry references and citations for amino-acids computed in this model. Ligand forms were taken from the PDB^75^, and zwitterionic and optimized geometries are taken from the CSD^35^. Values are in ppm-a.u. Cations are in **bold**, and anions are underlined. Values separated by commas indicate two indicated geometries in the same reference (e.g., two possible bond angles in the carboxyl group from the specified geometry). The maximum produced *ee*, *EE*_*max*_ is for a meteoroid with v/c = 0.015 in a 30 T field at T = 10 K and *f* = 5 × 10^−9^.

In Table 1, it is seen that nearly all zwitterionic forms have the same sign of *η*_*M*_. Also included in this table are the results for the zwitterionic forms after optimizing the literature values as described previously. For the optimized geometries nearly all values of *η*_*M*_ have the same sign, which would result in the same sign of the *ee* for each amino acid in a single meteoroid assuming the same environment throughout.

There are a few notable cases present in this table. Arginine and histidine both have *η*_*M*_ < 0 in the zwitterionic state. However, both of these amino acids are likely to have positively-charged side chains in solution. The values of *η*_*M*_ for these cationic states are shown to be positive. The chosen geometry may have significant uncertainty for these amino acids, however, as it is for the anhydrous monohydrate state, and multiple geometries were listed in the same reference.

Isovaline is another interesting case, as its non-optimized and optimized zwitterionic forms both have *η*_*M*_ < 0. There are few geometries available for isovaline in the literature, and those available are for isovaline monohydrate. However, in the cationic state, which may occur in hydrochloride solutions, *η*_*M*_ becomes quite large owing to the significant shift in the dipole moment. In this case, as no literature values for cationic isovaline were found, this form was adapted from the monohydrate value and optimized in aqueous solution using the same method as the other optimized values in the table. Additional studies of the geometry of isovaline in multiple forms would be welcome and important, as this amino acid was found to have a relatively large positive *ee* in the Murchison meteorite^9^.

With the evaluated values of *η*_*M*_, amino acids in Table 1 were simulated in a computer code following the prescription of reference^6^ assuming molecules embedded in meteoroid cores and processed in high magnetic fields by antineutrinos. Within the meteoroid, an initial population of equal parts D- and L- amino acids is assumed. The spin-lattice relaxation time for the nitrogen nucleus was assumed to be 10 ms, which is estimated from previous evaluations^36^. This time could also depend on temperature and crystallization state of the amino acid^37^. We assume that the antineutrino and meteoroid velocity vectors are co-linear.

In this model, one spin-chiral combination is preferentially destroyed (When the N is converted to C, the molecule is no longer an amino acid.), which defines the SNAAP model. From Famiano *et al*.^6^ (Fig. 6) it is seen that the antineutrino spin alignment with the parallel (*N*_+_) nitrogen spin for the L- amino acid is less than 90° (with respect to the meteoroid velocity vector) while it is greater than 90° in the D- amino acid. The D- amino acid is preferentially destroyed in this configuration as there is a larger spin component opposite the antineutrino spin. For meteoroids moving toward (away from) the central object, negative (positive) *ee*s are produced.

The interaction rate for antineutrino spins parallel to the nitrogen spin is parametrized as a factor relative to the inverse relaxation time *R*_*p*_ = *f*/(2*T*_1_). This is the model’s only free parameter and varies with the environment in which the model occurs. At a distance of 1 AU from a type II SN, *f* ~ 10^−9^ ^38^. For a neutron star merger *f* can be 10^−4^ or much larger depending on meteoroid proximity^39^. Cooling neutron stars may present another scenario. In this case, *f* ~ 10^−10^ to 10^−8^ ^40^. The antineutrino reaction rate is expected to be much smaller for cooling neutron stars. Despite this, the flux of antineutrinos could last for at least 10^5^ years. Processing is possible as long as the magnetic field is present. If the meteoroid is in the vicinity of the neutron star for a few months or years, the amino acids will be slowly processed. Amino acids near a neutron star merger are expected to be rapidly processed. Any situation with an antineutrino flux and high magnetic fields can result in amino acid processing. Equation 5 is then used to estimate the relative rates of amino acid destruction for each spin configuration. We also note that the average nuclear spins are nearly perpendicular to the velocity vectors of the antineutrinos.

**Figure 1:**
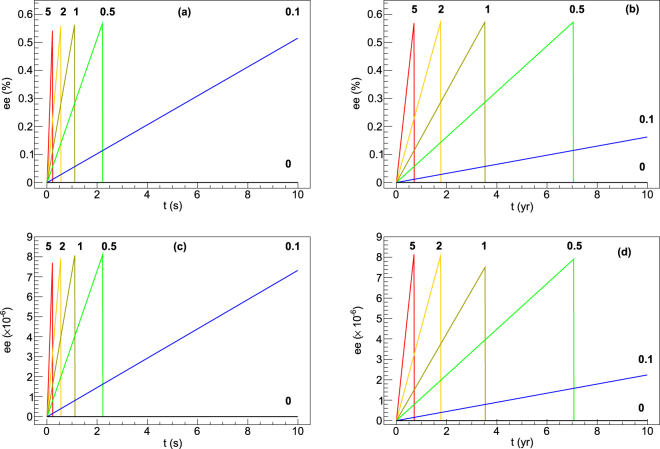
The isovaline cation *ee* as a function of time for a meteoroid penetrating the external magnetic field for scenarios described in the text, (**a**) B = 800 T with high f, (**b**) B = 800 T with low f, (**c**) B = 30 T with high f, and (c) B = 30 T with low f. Values of f are indicated beside the lines. In all cases v = 0.015c. For plots (b) and (d) the values of f should be multiplied by 10^−8^.

Figure 1 shows the results of this calculation for cationic isovaline for four scenarios. These scenarios show four situations for high and low magnetic fields and high and low antineutrino rates. The cooling neutron star scenario could be represented by a magnetic field of 800 T and a meteoroid velocity of 0.015c. For these calculations, the core temperature of the asteroid was assumed to be 10 K. Antineutrino reaction rate ratios for this situation are *f* = 0, 10^−9^, 5 × 10^−9^, 10^−8^, 2 × 10^−8^, and 5 × 10^−8^. A high-rate, low-field scenario simulates a meteoroid in proximity to a neutron star merger. Here *f* = 0, 0.1, 0.5, 1, 2, and 5; antineutrino rates that span a large range relative to the relaxation rate. In this scenario, a magnetic field of 30 T and a velocity of 0.015c is assumed. The produced *ee* as a function of time is shown in each plot starting from an *ee* of zero. In this model the antineutrino flux controls the rate at which the *ee* changes while the field strengths control the maximum *ee* possible. Of course, for no external antineutrino interactions (*f* = 0), the *ee* does not change. For all models, the number of molecules within a chiral state remains unchanged.

In addition to type II supernova and neutron star mergers, neutron star cooling from a binary star system consisting of a neutron star and a Wolf-Rayet star^17^ may present a possible scenario. With external magnetic fields of 800 T at a distance of 500 km and a velocity of 0.015c, the *ee*s can be as high as ~1%. This distance is well within the range of the accretion disk size for a neutron star^17,41,42^. It is likely that the meteoroid is in an elliptic orbit, so speed would be non-constant. Additionally, disk viscosity could impact the speed. However, for a circular orbit at 500 km, the classical orbital velocity would be 0.05c. Thus, we choose a conservative average speed of 0.015c.

The *ee* will eventually drop to zero if the meteoroid spends too much time in the antineutrino flux as all the amino acids will be destroyed. A positive *ee* can be produced for any period of time between 0 and the time at which the *ee* is a maximum. For a cooling neutron star with a very high field and low antineutrino flux the time to a maximum *ee* is months to years, the processing time of the meteoroid prior to ejection from the system. Amino acids will be processed over multiple orbits. A non-zero *ee* can be produced during the meteoroid’s exposure to the antineutrino flux.

**Figure 2:**
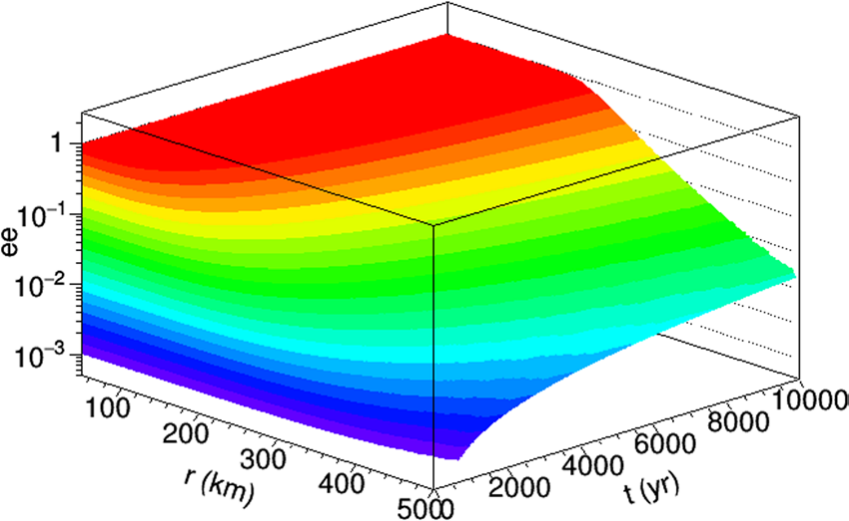
The *ee* produced in zwitterionic alanine orbiting a neutron star for various average radii and times.

Because of the wide range in environmental parameters available to this mode, we provide a representation of the *ee* for zwitterionic alanine as a function of time and distance from a neutron star in the NS-WR binary system in Fig. 2. In this system, the meteoroid is presumed to orbit a neutron star at the average radius indicated on the plot for the amount of time indicated on the plot. In this figure, the meteoroid velocity is computed as a function of radius about a neutron star with a surface field of 10^15^ G, a mass of 1 ${{\rm{M}}}_{\odot }$, and an antineutrino flux of 10^38^ s^−1^. Thus, one can use this figure to determine the *ee* for alanine for an orbit for a certain amount of time.

The maximum *ee*s computed in this model are approximately proportional to *η*_*M*_ from Table 1. Thus, we can make a comparison to the relative production of amino acid *ee*s in our model to those found in the Murchison and Murray meteorites^43^. We show the ratios *η*_*M*_/*η*_*M*,*ALA*_ in our model compared to the ratios *ee*/*ee*_*ALA*_ found in these meteorites^12^ in Table 2. Data from three sources - CI1 (from the Orgueil meteorite), the CM2 (from the Murchison meteorite), and CR3 (from the Antarctic meteorite QUE 99177) - samples were examined. The CI1 source is known to have a higher degree of aqueous alteration than the CM2 sample, which has a higher level of aqueous alteration than than the CR3 sample. In the case of glutamic and aspartic acids, the optimized zwitterion geometries were used (though these are generally thought to be anionic in solution). For glutamic acid, the value for the zwitterion is very similar to that of the anion, while for aspartic acid, the zwitterionic ratio is twice what it would be for the anionic state. Negative values indicate *ee*s with a sign opposite to those of alanine using the molecular geometries in Table 1. We see that the presumed ligand and zwitterion computed *ee* values are not consistent with observations. However, after optimizing these geometries for production in aqueous solutions, the produced *ee*s are closer to those found in the Murchison meteorite^13^, with values that appear to more closely match those of the CI1 sample, with the exception of glutamic acid, with a lower value. It seems that an aqueous solution is necessary during or prior to processing. Certainly the geometry is important in considering this production. Also, amino acids bound to larger molecules may exhibit shifts in *η*_*M*_ as well. Further, the limited geometries available for isovaline may be relevant as the geometry used here was for crystalline isovaline monohydrate, which we subsequently optimized in aqueous solution. More study is warranted for this molecule.

**Table 2:** Ratios of the geometry factor for three amino acids (in %) to that of alanine in this model compared to the equivalent ratios of *ee*s found in the meteoric samples with the most aqueous alteration (CI1), the least aqueous alteration (CR3), and an intermediate amount of aqueous alteration (CM2)^12^.

	Ligand	Zwitterion	Optimized Cation	CI1	CM2	CR3
Isovaline	0.16	−0.06	2.20	1.92 (0.16)	2.45 (0.16)	0.05 (0.14)
Norvaline	−1.42	0.83	0.20	0.45 (0.17)	0.0 (0.14)	0.05 (0.14)
Aspartic Acid*	−2.90	0.37	0.60	0.12 (0.17)	2.00 (0.15)	−0.10 (0.15)
Glutamic Acid*	−0.24	0.52	0.73	3.38 (0.17)	2.45 (0.17)	−0.10 (0.18)

Values in parentheses are uncertainties estimated from computed error propagation from the uncertainties provided with the original data.

*Optimized zwitterion geometries used in place of the optimized cation.

Here, we point out that - in many cases - the uncertainties in the measurements can be large. We also note that the range of values between the two samples is quite large. However, the predicted values are similar to the mean *ee*s observed.

Any differences may be due to differences between how the amino acids are processed in the SNAAP model and any subsequent post-processing through aqueous alteration^12^, racemization, heating, and solution effects. In the current model, the amino acids are assumed to be racemic prior to processing. Processing takes a very short amount of time compared to any subsequent racemization and delivery. Because amino acids have different racemization times, subsequent processing of the meteorite will alter the initial ratios^44^. This has been used in the past as a dating mechanism. Given the variability in racemization times with environment, perhaps it is possible to infer meteoric age and subsequent environmental processing in this model.

Cumulative environmental effects (e.g., pH, temperature, solvent, etc.) were accounted for in the current study by assuming specific geometries. Amino acid geometries were taken from the CSD database where appropriate^35^. For geometry optimization, the geometry references indicated in Table 1 were optimized assuming a neutral water solution with the Polarizable Continuum Model (PCM)^45^. This was independent of any possible subsequent freezing or processing. Obviously, this is a simplification, and future work will concentrate on more accurate geometry effects. Thus, care is warranted in comparing values in Table 2.

Finally other effects may shift values produced in this model after processing. Subsequent racemization - which occurs at different rates for different amino acids - and auto-catalysis have not been considered in our present model. Other effects may also impact the results. Complex trajectories of the meteoroids may affect the *ee*s, as well as inclusion of the time changing magnetic fields they will encounter. More amino acids may exist in anionic or cationic states, and this may also affect their *ee*s. Further studies will concentrate on more realistic trajectories as well as the interior environment of the body containing the amino acids. None the less, the SNAAP model presents a possibility for production of amino acid *ee*s that are approaching the range of those observed in meteorites.

## Methods

The detailed method used to produce the results in this paper are described in reference^6^ and appendix A therein.

While the DALTON code^46^ was used in reference^6^, the Gaussian^27^ code was used to produce the results in this work using the computational method and basis sets described in the text.

### Data availability

The datasets generated during and/or analysed during the current study are available from the corresponding author on reasonable request.
